# “I must be perfect”: The role of irrational beliefs and perfectionism on the competitive anxiety of Hungarian athletes

**DOI:** 10.3389/fpsyg.2022.994126

**Published:** 2022-09-20

**Authors:** Renátó Tóth, Martin J. Turner, Tibor Kökény, László Tóth

**Affiliations:** ^1^School of Doctoral Studies, Hungarian University of Sports Science, Budapest, Hungary; ^2^Faculty of Health and Education, Manchester Metropolitan University, Manchester, United Kingdom; ^3^FTC Handball Academy, Budapest, Hungary; ^4^Department of Psychology and Sport Psychology, Hungarian University of Sports Science, Budapest, Hungary

**Keywords:** irrational beliefs, competitive anxiety, rational emotive behavioral therapy, adaptive perfectionism, maladaptive perfectionism

## Abstract

In this study the influence of irrational beliefs and perfectionism on the emergence of competitive anxiety was investigated. While previous studies indicate that higher irrational beliefs predict greater competitive anxiety, in the present study it is hypothesized that this relationship is mediated by perfectionism. A serial atemporal multiple mediation analysis revealed that both adaptive and maladaptive perfectionism were significant partial mediators between irrational beliefs and competitive anxiety. The total score and all four subscale scores on irrational beliefs had both direct and indirect effects on cognitive competitive anxiety, the latter effects mediated by both forms of perfectionism. Depreciation beliefs had a direct effect, and demandingness and awfulizing had indirect effects, on somatic competitive anxiety when both forms of perfectionism were entered as mediators. These findings suggest that both irrational beliefs and perfectionism influence the emergence of competitive anxiety, therefore rational emotive behavioral therapy with a focus upon perfectionism may be an effective means of reducing competitive anxiety in athletes.

## Introduction

Sport is a highly competitive field in which athletes are required to meet high expectations and be both physically and mentally fit to reach peak performance. More and more sport professionals recognize the importance of mental preparation in optimizing performance and maintaining mental health. For example, nineteen-time US gymnastics world champion Simone Biles withdrew from the finals of the 2020 Tokyo Olympic Games in order to focus on her wellbeing (Ingle, 2021). As three-time Olympic champion Hungarian fencer Áron Szilágyi noted shortly after winning his third Olympic title, “sport is a universal phenomenon, and I do not think there is a sport in the world where revolutionary innovations are yet to be discovered in terms of preparation, tactics or even technique. In short, they have maximized what they had, so the importance of mental preparation has increased dramatically because that is where there are still untapped advantages to be gained” (Molcsapat, 2022).

One of the most common targets in athletes’ mental screening and preparation is competitive anxiety, which is a negatively valenced emotional state that could lead to drastic performance reduction under pressure if not managed properly (Hardy, 1990), although whilst anxiety is often experienced as an unpleasant emotional state, it is not always maladaptive (Ellis and DiGiuseppe, 1993) or related to poor performance (Craft et al., 2003). Several theoretical models have emerged from studies of the relationship between sport performance and anxiety. The most popular theory is Martens et al. (1990) multidimensional anxiety model, which makes a distinction between three major factors of competitive anxiety: cognitive anxiety, somatic anxiety, and self-confidence. Cognitive anxiety involves concerns about, and negative expectations for performance, while somatic anxiety includes physical symptoms (e.g., wet palms, muscle tension, and increased heart rate). Both high and low levels of somatic anxiety may impair sport performance, since high somatic anxiety leads to the mentioned autonomic symptoms, while low somatic anxiety is associated with fatigue, boredom, lethargy, etc. While this model offers an explanation for the various symptoms of anxiety in athletes, it is still not fully clarified what psychological factors influence the emergence of competitive anxiety.

Recently, increasing attention has been focused on the benefits of cognitive behavior therapies (CBTs) in sport psychology (e.g., Turner et al., 2020a), including Ellis (1999) rational emotive behavioral therapy (REBT). The adaptation of REBT for sport-related objectives is a relatively new research area (Turner, 2016) but research and professional practice literature us growing (Jordana et al., 2020). The theoretical model undergirding REBT primarily focuses on rational (flexible, non-extreme, and logical) and irrational (rigid, extreme, illogical) beliefs. Four forms of irrational beliefs are distinguished. Dogmatic demandingness (so-called primary irrational beliefs) includes maladaptive demands on one’s own performance, illustrated by statements such as *I want to be successful, and that means I have to be successful*. The model makes a distinction between three types of secondary irrational beliefs such as awfulizing (*If I don’t succeed, it will be terrible*), low frustration tolerance (*failure is unbearable to me*), and depreciation (*If I fail, I am a complete failure*; Dryden, 1995). These Irrational beliefs have been found to be associated with trait, state, social, and performance anxiety in both clinical and non-clinical samples (Himle et al., 1982; Deffenbacher et al., 1986; Wood et al., 2018), and in specific athlete samples (e.g., Chadha et al., 2019; Turner et al., 2019a, 2022; Mansell, 2021). Browne et al. (2010) enumerated a number of unhealthy factors associated with irrational beliefs including anger, guilt and shame, and psychopathological constructs such as depression, anxiety and suicidal thoughts. A more recent meta-analysis revealed relatively high correlations between irrational beliefs and general distress, depression, anxiety, anger, and guilt (Visla et al., 2016). Also, various applied studies that utilize REBT have indicated that as irrational beliefs reduce, so too do forms of anxiety (e.g., Turner and Barker, 2013; Turner et al., 2020b; Bowman and Turner, 2022), intimating a connection between irrational beliefs and anxiety.

In REBT theory and practice, greater irrational beliefs have been linked to greater perfectionism (Flett et al., 1991; Ellis, 2002). Perfectionism involves excessively high expectations of oneself followed by critical self-evaluation of performance (Frost et al., 1990). There is irrationality nested or implied within the notion of perfectionism, because the idea that something (especially a human being, or the outcomes driven by human beings) could be perfect is an extreme and illogical idea. As fallible and ever-changing human beings, we are not capable of perfection. However, outside of REBT, perfectionism is considered to be a multidimensional personality characteristic that can be both adaptive and maladaptive (Gotwals et al., 2012). Several dimensions of perfectionism have been identified in sport contexts, which are currently divided into the two broad categories of adaptive vs. maladaptive perfectionism (strivings vs. concerns; Gotwals et al., 2012). Perfectionist strivings include one’s internal standards and drives toward perfection. Perfectionist concerns (expectations) are associated with fear of failure and negative reactions to imperfection (Stoeber and Otto, 2006).

Perfectionism has long been considered as a factor that can be detrimental to sport performance, since perfectionist expectations are potentially associated with negative cognitive, emotional and behavioral consequences (Limburg et al., 2017). However, this does not equally apply for both forms of perfectionism. Perfectionist strivings in sport have been found to be associated with positive emotions (Sagar and Stoeber, 2009), stable self-confidence, and low cognitive and somatic competitive anxiety (Stoeber et al., 2007). However, Gotwals and Tamminen (2022) could not consistently distinguish between adaptive and maladaptive perfectionism in various situational contexts, since the relevance of the distinction also depended on other psychological factors such as athletes’ susceptibility to irrational beliefs, which was associated with increased distress after negative events (failure). These findings are in line with the alternative distinction between *standards* and *expectations* (Rice and Ashby, 2007), and with the finding that adaptive perfectionism potentially enhances athletic performance, while maladaptive perfectionism is negatively associated with mental health (Hill et al., 2018). Nevertheless, the previous findings have not clarified whether perfectionism has a positive or negative influence on athletes’ mental health. As Hill et al. (2020) point out, any form of perfectionism, despite its positive effects, can pose a threat to one’s wellbeing. If an athlete is perfectionistic and holds irrational beliefs, it is surely likely that they will suffer psychological distress, including chronic or acute anxiety.

The present study explores the associations between irrational beliefs, perfectionism, and competitive anxiety in a sample of athletes. While previous findings have shown that irrational beliefs influence the emergence of competitive anxiety, studies that have examined the relationship between perfectionism (primarily strivings) and competitive anxiety have not revealed a consistently positive or negative association between the two constructs. Separately, the influence of irrational beliefs and perfectionism on competitive anxiety has been demonstrated, but the relationship between irrational beliefs and perfectionism, and their combined effect upon anxiety, has not been investigated in athletes. Hence, in the present study we explore the associations between these three variables utilizing atemporal mediational models. It is hypothesized that irrational beliefs would have significant indirect effects on cognitive and somatic competitive anxiety, mediated by adaptive and maladaptive perfectionism (standards and discrepancy, respectively). In other words, we anticipate that it is through perfectionism that irrational beliefs exert the most impact upon anxiety.

## Materials and methods

### Participants and procedure

The sample consisted of 219 Hungarian athletes (123 males, 96 females, *M* = 26.39), who were invited e-mail *via* various sports clubs to complete an online form. The sample included both recreational (*N* = 140) and professional athletes (*N* = 79) pursuing diverse individual (*N* = 82) and team sports (*N* = 137), including boxing, speed skating, soccer, ice hockey, handball, and table tennis. The studies involving human participants were reviewed and approved by Hungarian University of Sport Science Research Ethics Committee. Written informed consent to participate in this study was provided.

### Measures

Competitive state anxiety was measured with the Competitive State Anxiety Inventory-2 (CSAI-2; Martens et al., 1990; Hungarian adaptation by Sipos et al., 1999). The questionnaire contains 27-items divided into three scales assessing cognitive anxiety, somatic anxiety, and self-confidence. The Hungarian version demonstrated high internal consistency (Cronbach’s αs ranged from 0.75 to 0.85) (see Table 1).

**TABLE 1:** Descriptive statistics, reliability, and correlation of variables (iPBI, SAPS, and CSAI-2-H).

	M	SD	Cronbach-alpha	DEM	LFT	AWF	DEP	IBs	STD	DCY	CAN	SAN
DEM	3.64	0.77	0.70	–	0.35**	0.68**	0.30**	0.71**	0.22**	0.12	0.18**	0.15*
LFT	3.84	0.85	0.84	0.35**	–	0.55**	0.56**	0.79**	0.58**	0.48**	0.37**	0.27**
AWF	3.61	0.89	0.77	0.68**	0.55**	–	0.53**	0.87**	0.35**	0.27**	0.31**	0.17*
DEP	2.60	0.93	0.79	0.30**	0.57**	0.53**	–	0.78**	0.30**	0.52**	0.49**	0.36**
IBs	3.42	0.68	0.89	0.72**	0.79**	0.87**	0.78**	–	0.46**	0.45**	0.44**	0.29**
STD	5.92	0.96	0.83	0.22**	0.58**	0.35**	0.30**	0.46**	–	0.49**	0.27**	0.17*
DCY	4.35	1.44	0.81	0.12	0.48**	0.27**	0.52**	0.45**	0.49**	–	0.46**	0.22**
CAN	2.29	0.62	0.83	0.18**	0.37**	0.31**	0.48**	0.44**	0.27**	0.46**	–	0.67**
SAN	2.01	0.53	0.75	0.15*	0.22**	0.17*	0.36**	0.29**	0.17*	0.22**	0.67**	–

**p < 0.01, *p < 0.05. DEM, demandingness; LFT, low-frustration tolerance; AWF, awfulizing; DEP, depreciation; IBs, iPBI total; STD, standards (adaptive perfectionism); DCY, discrepancy (maladaptive perfectionism); CAN, cognitive anxiety; SAN, somatic anxiety.

Irrational beliefs were measured with the Irrational Performance Beliefs Inventory (iPBI; Turner and Allen, 2018). The iPBI contains 20-items composing the four subscales of demandingness (DEM), awfulizing (AWF), low frustration tolerance (LFT), and depreciation (DEP). The original instrument showed adequate validity and test-retest reliability (Turner and Allen, 2018). In the absence of a previous Hungarian adaptation, a Hungarian translation of the iPBI (e.g., Chotpitayasunondh and Turner, 2019; Nejati et al., 2022) was back-translated into English by an independent native English-speaking colleague, and the authors of the original instrument found the back-translation acceptable, thus construct equivalence was ensured. As the first table shows all subscales of the Hungarian version showed adequate internal consistency (Cronbach’s αs ranged from 0.70 to 0.89).

Trait-level adaptive and maladaptive perfectionism (i.e., standards and discrepancy, respectively) were measured with the eight-item revised short Almost Perfect Scale (SAPS; Rice et al., 2014; Hungarian adaptation by Reinhardt et al., 2018). The standards subscale (four items) assesses factors related to high performance expectations, while the discrepancy subscale (four items) assesses the perceived distance from self-set standards and related concerns. The Hungarian version showed adequate reliability and validity, and no significant correlation was found between the two subscales (Reinhardt et al., 2018, 2019). The reliability of the questionnaire is also appropriate for the current sample (Cronbach’s αs ranged from 0.81 to 0.83) (see Table 1).

### Statistical data analyses

**FIGURE 1:**
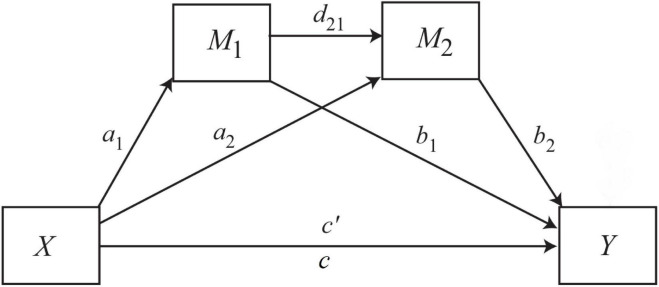
Serial atemporal multiple mediation model with two mediators. X = independent variable; Y = dependent variable; M_1_, M_2_ = mediators; a_1_, a_2_, b_1_, b_2_, d2_1_, c, c′ = regression coefficients.

All measures were first checked for normality, and all showed normal distribution. The descriptive statistics and Pearson intercorrelations of the measures were then calculated using the IBM SPSS program. The direct and indirect effects of irrational beliefs on competitive anxiety with perfectionism competitive anxiety as a mediator were tested with serial multiple mediation (SAMM) analyses using the PROCESS macro v. 4.10 integrated into the IBM SPSS program. We ran Hayes (2022) model 6, 10 times, since we had two mediators (standards, discrepancy) in each case (see Figure 1). The independent variables (X) were the iPBI total score (irrational beliefs) and its four subscale scores, while the dependent variables (Y) were cognitive and somatic anxiety (as measured with the CSAI-2-H) and the mediators was the two form of perfectionism (adaptive and maladaptive) standards (M_1_) and discrepancy (M_2_) which measured by SAPS.

## Results

### Descriptive statistics and intercorrelations

The descriptive statistics and intercorrelations of the target variables are presented in Table 1. All measures of irrational beliefs correlated positively with both cognitive and somatic anxiety and with both adaptive and maladaptive perfectionism. Furthermore, both forms of perfectionism correlated positively with both cognitive and somatic anxiety.

### Serial atemporal multiple mediation analyses

A total of 10 serial atemporal multiple mediation (SAMM) analyses were conducted to test the direct and indirect effects of irrational beliefs on competitive anxiety (cognitive and somatic) through perfectionism (adaptive and maladaptive). The results are presented in Table 2. The total effect of irrational beliefs on competitive anxiety was significant in each of the 10 mediation models.

**TABLE 2:** Serial atemporal multiple mediation analysis results.

Model no.	X	M1	M2	Y	YR^2^	Total c (95% CI)	Direct c’ (95% CI)
					F(,)	t(df)	t(df)
					*p*	*p*	*p*
1	DEM	STD	DCY	CAN	0.23	0.15	0.10
					*F*(3, 215) = 21.18	*t*(218) = 2.71	*t*(218) = 2
					*p* < 0.001	*p* = 0.007	*p* = 0.04
2	AWF	STD	DCY	CAN	0.25	0.21 (0.13–0.30)	0.14 (0.001–0.22)
					*F*(3, 215) = 23.53	*t*(218) = 4.74	*t*(218) = 3.01
					*p* < 0.001	*p* < 0.001	*p* = 0.02
3	LFT	STD	DCY	CAN	0.24	0.27 (0.18–0.36)	0.15 (0.04–0.26)
					*F*(3, 215) = 22.42	*t*(218) = 5.79	*t*(218) = 2.63
					*p* < 0.001	*p* < 0.001	*p* = 0.009
4	DEP	STD	DCY	CAN	0.30	0.33 (0.25–0.41)	0.23 (0.14–0.32)
					*F*(3, 215) = 30.83	*t*(218) = 8.36	*t*(218) = 5.17
					*p* < 0.001	*p* < 0.001	*p* < 0.001
5	IBS	STD	DCY	CAN	0.28	0.40 (0.29–0.51)	0.27 (0.15–0.39)
					*F*(3, 215) = 27.42	*t*(218) = 7.11	*t*(218) = 4.33
					*p* < 0.001	*p* < 0.001	*p* < 0.001
6	DEM	STD	DCY	SAN	0.06	0.10 (0.009–0.19)	0.08 (−0.01 to 0.17)
					*F*(3, 215) = 4.92	*t*(218) = 2.17	*t*(218) = 1.66
					*p* = 0.003	*p* = 0.03	*p* = 0.10
7	AWF	STD	DCY	SAN	0.06	0.10 (0.02–0.18)	0.07 (−0.02 to 0.15)
					*F*(3, 215) = 4.81	*t*(218) = 2.58	*t*(218) = 1.58
					*p* = 0.003	*p* = 0.01	*p* = 0.12
8	LFT	STD	DCY	SAN	0.25	0.13 (0.05–0.22)	0.09 (−0.02 to 0.19)
					*F*(3, 215) = 4.88	*t*(218) = 3.26	*t*(218) = 1.64
					*p* = 0.003	*p* = 0.001	*p* = 0.10
9	DEP	STD	DCY	SAN	0.13	0.20 (0.13–0.27)	0.19 (0.11–0.27)
					*F*(3, 215) = 11.01	*t*(218) = 5.68	*t*(218) = 4.48
					*p* < 0.001	*p* < 0.001	*p* < 0.001
10	IBS	STD	DCY	SAN	0.09	0.22 (0.12–0.32)	0.18 (0.07–0.30)
					*F*(3, 215) = 7.34	*t*(218) = 4.43	*t*(218) = 3.11
					*p* < 0.001	*p* < 0.001	*p* = 0.002

DEM, demandingness, DEP, depreciation; LFT, low-frustration tolerance; AWF, awfulizing; IBS, irrational beliefs total; STD, standards (adaptive perfectionism); DCY, discrepancy (maladaptive perfectionism); CAN, cognitive anxiety; SAN, somatic anxiety.

#### Relationships between irrational beliefs, perfectionism, and cognitive anxiety

**FIGURE 2:**
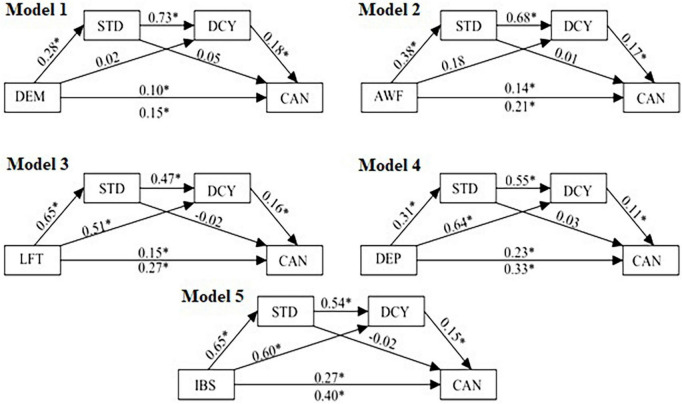
Regression weights for the SAMM models with irrational beliefs (iPBI; DEM, AWF, LFT, DEP, IBS), perfectionism (SAPS; STD, DCY) and cognitive anxiety (CSAI-2; CAN). *p < 0.05. DEM, demandingness; LIT, low-frustration tolerance; AWF, awfulizing; DEP, depreciation; IBS, total of irrational beliefs; STD, standards (adaptive perfectionism); DCY, discrepancy (maladaptive perfectionism); CAN, cognitive anxiety.

Significant positive direct effects were obtained for demandingness (β = 0.27, 95% CI = 0.001–0.20), awfulizing (β = 0.14, 95% CI = 0.05–0.22), low frustration tolerance (β = 0.15, 95% CI = 0.04–0.26), depreciation (β = 0.23, 95% CI = 0.14–0.32), and total irrational beliefs (β = 0.27, 95% CI = 0.15–0.39) with cognitive anxiety as the outcome variable and both standards and discrepancy entered as mediators (see Table 2). Importantly, adaptive perfectionism (standards) alone did not significantly predict cognitive anxiety in any of the SAMM models (see Figure 2). The indirect effects of demandingness (β = 0.04, 95% CI = 0.1–0.07), awfulizing (β = 0.06, 95% CI = 0.03–0.10), low frustration tolerance (β = 0.07, 95% CI = 0.02–0.03), depreciation (β = 0.03, 95% CI = 0.01–0.05), and total irrational beliefs (β = 0.06, 95% CI = 0.03–0.09) on cognitive anxiety were significant only when both mediators (standards and discrepancy) were entered (see Table 3). Significant indirect effects were obtained for low frustration tolerance (β = 0.11, 95% CI = 0.03–0.05), depreciation (β = 0.11, 95% CI = 0.04–0.17), and total irrational beliefs (β = 0.10, 95% CI = 0.03–0.09) with cognitive anxiety as the outcome variable and discrepancy as the mediator.

**TABLE 3:** Indirect effects of serial atemporal multiple mediations (SAMM).

Model 1					Indirect effect (95% CI)
Ind1	DEM	STD	CAN		0.01 (−0.02 to 0.03)
Ind2	DEM	DCY	CAN		0.003 (−0.04 to 0.05)
**Ind3**	**DEM**	**STD**	**DCY**	**CAN**	**0.04 (0.01 to 0.07)**
Model 2					Indirect effect (95% CI)
Ind1	AWF	STD	CAN		0.02 (−0.05 to 0.51)
Ind2	AWF	DCY	CAN		0.04 (−0.003 to 0.10)
**Ind3**	**AWF**	**STD**	**DCY**	**CAN**	**0.06 (0.03 to 0.10)**
Model 3					Indirect effect (95% CI)
Ind1	LFT	STD	CAN		−0.02 (−0.11 to 0.07)
**Ind2**	**LFT**	**DCY**	**CAN**		**0.11 (0.03–0.05)**
**Ind3**	**LFT**	**STD**	**DCY**	**CAN**	**0.07 (0.02–0.03)**
Model 4					Indirect effect (95% CI)
Ind1	DEP	STD	CAN		0.01 (−0.03 to 0.05)
**Ind2**	**DEP**	**DCY**	**CAN**		**0.11 (0.04–0.17)**
**Ind3**	**DEP**	**STD**	**DCY**	**CAN**	**0.03 (0.01–0.05)**
Model 5					Indirect effect (95% CI)
Ind1	IBS	STD	CAN		−0.01 (−0.08 to 0.05)
**Ind2**	**IBS**	**DCY**	**CAN**		**0.10 (0.03–0.09)**
**Ind3**	**IBS**	**STD**	**DCY**	**CAN**	**0.06 (0.03–0.09)**
Model 6					Indirect effect (95% CI)
Ind1	DEM	STD	SAN		0.01 (−0.03 to 0.05)
Ind2	DEM	DCY	SAN		0.002 (−0.02 to 0.03)
**Ind3**	**DEM**	**STD**	**DCY**	**SAN**	**0.02 (0.001–0.05)**
Model 7					Indirect effect (95% CI)
Ind1	AWF	STD	SAN		0.02 (−0.04 to 0.07)
Ind2	AWF	DCY	SAN		0.02 (−0.003 to 0.05)
**Ind3**	**AWF**	**STD**	**DCY**	**SAN**	**0.03 (0–0.06)**
Model 8					Indirect effect (95% CI)
Ind1	LFT	STD	SAN		0.008 (−0.09 to 0.10)
Ind2	LFT	DCY	SAN		0.04 (−0.01 to 0.10)
Ind3	LFT	STD	DCY	SAN	0.03 (−0.01 to 0.07)
Model 9					Indirect effect (95% CI)
Ind1	DEP	STD	SAN		0.02 (−0.03 to 0.06)
Ind2	DEP	DCY	SAN		0.01 (−0.07 to 0.08)
Ind3	DEP	STD	DCY	SAN	0.002 (−0.02 to 0.02)
Model 10					Indirect effect (95% CI)
Ind1	IBS	STD	SAN		0 (−0.02 to 0.13)
Ind2	IBS	DCY	SAN		0.02 (−0.01 to 0.07)
Ind3	IBS	STD	DCY	SAN	0.01 (−0.01 to 0.04)

Variables in bold indicate significant SAMM paths. DEM, demandingness; DEP, depreciation; LFT, low-frustration tolerance; AWF, awfulizing; IBS, total of irrational beliefs; STD, standards (adaptive perfectionism); DCY, discrepancy (maladaptive perfectionism); CAN, cognitive anxiety; SAN, somatic anxiety.

#### Serial atemporal multiple mediation among irrational beliefs, perfectionism, and somatic anxiety

**FIGURE 3:**
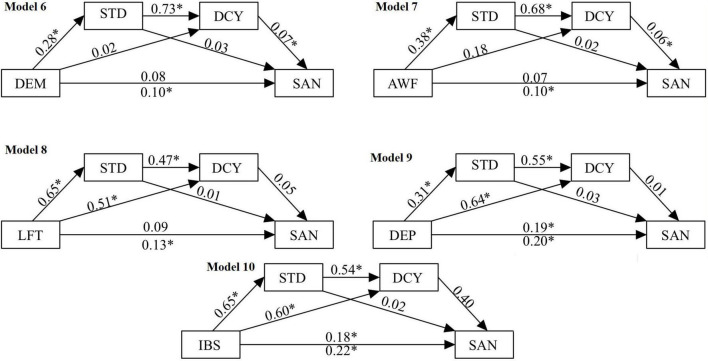
Regression weights for the SAMM models with irrational beliefs (iPBI), perfectionism (SAPS) and somatic anxiety (CSAI-2). *p < 0.05. DEM, demandingness, LFT, low-frustration tolerance; AWF, awfulizing; DEP, depreciation; IBS, total of irrational beliefs; STD, standards (adaptive perfectionism); DCY, discrepancy (maladaptive perfectionism); SAN, somatic anxiety.

Significant positive direct effects were obtained for depreciation (β = 0.19, 95% CI = 0.11–0.27), and total irrational beliefs (β = 0.18, 95% CI = 0.07–0.30) with somatic anxiety as the outcome variable and both standards and discrepancy entered as mediators (see Figure 3). Adaptive perfectionism (standards) did not significantly predict somatic anxiety in any of the tested models. Demandingness (β = 0.02 95% CI = 0.01–0.05) and awfulizing (β = 0.03, 95% CI = 0.00–0.06) had significant indirect effects on somatic anxiety when both mediators (standards and discrepancy) were entered.

## Discussion

In the present study we explored the effects of irrational beliefs on competitive anxiety through adaptive (standards) and maladaptive (discrepancy) perfectionism. To the authors’ knowledge, this study is the first to explore the effects of irrational beliefs on competitive anxiety among Hungarian athletes, and it is the first to test adaptive and maladaptive perfectionism as mediators of the mentioned effects. It also adds to and extends the limited research that has linked irrational beliefs to perfectionism.

### Effects of irrational beliefs on competitive anxiety through perfectionism

Both primary and secondary irrational beliefs were positively associated with both cognitive and somatic competitive anxiety in athletes, which corroborates previous related findings (e.g., Chadha et al., 2019). Adaptive and maladaptive perfectionism were also positively associated with both forms of competitive anxiety, in line with the findings of Hill et al. (2020), who emphasize that either form of perfectionism can pose a threat to one’s wellbeing. However, the positive correlations between adaptive perfectionism and competitive anxiety were low in magnitude, thus they are not inconsistent with the finding that perfectionist standards in sport may in some cases be associated with positive emotions and low cognitive and somatic competitive anxiety (Stoeber et al., 2007; Sagar and Stoeber, 2009). These results suggest that there is a relationship between athletes’ irrational beliefs and trait-level perfectionism and they raise the possibility that perfectionism mediates the effects of irrational beliefs on perfectionism. As such, it appears that athletes who hold both strong irrational beliefs and strong perfectionistic beliefs could be a greater risk of experiencing higher competitive anxiety, but more technically, it is through perfectionistic beliefs that irrational beliefs might be more problematic.

Overall, the findings of the present paper indicate that athletes who hold stronger rigid, extreme and illogical thinking (irrational beliefs) are more concerned about their performance and possible negative consequences (cognitive anxiety), which is in large part due to their excessive expectations for, and highly critical attitude toward their own performance. Both irrational beliefs and perfectionistic beliefs reflect illogical and extreme ways of thinking, and as such, it is perhaps unsurprising that both are related to higher anxiety. Specifically, depreciation and low frustration tolerance had significant indirect effects on athletes’ cognitive anxiety when maladaptive perfectionism was the only mediator. Irrational beliefs also influenced the athletes’ somatic anxiety. Full mediation was found for the effects of demandingness and awfulizing with both forms of perfectionism entered as mediators (i.e., the indirect effects but not the direct effects were significant). This suggests that athletes with dogmatic self-set standards and fear of poor performance show higher somatic anxiety due to their perfectionist traits. Overall irrational beliefs, low frustration tolerance, and depreciation had significant direct effects but no indirect effect on somatic anxiety. That is, athletes who have difficulty tolerating adversity (LFT) and whose global sense of worth is highly vulnerable to, and in part defined by, momentary poor performance (DEP) are likely to experience higher levels of somatic anxiety irrespective of whether or not they have perfectionist standards and concerns. Importantly, adaptive perfectionism (standards) alone did not significantly mediate the effects of irrational beliefs on competitive anxiety in any of the tested models, which is consistent with the findings reported by Hill et al. (2018). These findings suggest that adaptive perfectionism may not have an antagonistic effect over above irrational beliefs, unlike maladaptive perfectionism alone, which did mediate between irrational beliefs and anxiety.

In sum, the obtained findings support the hypothesis that irrational beliefs positively predict competitive anxiety, and especially cognitive anxiety, through perfectionism, especially maladaptive perfectionism. Perfectionism also mediated the effects of two types of irrational beliefs (DEM and AWF) on somatic anxiety, a possible explanation for which is that these two types of irrational beliefs may be conceptually similar to perfectionistic beliefs. Overall, athletes who set dogmatic standards for themselves are not able to manage frustration and tend to awfulizing and disparagement themselves and others experience greater competitive anxiety. The following process influenced by perfectionist traits such as overly high expectations and a disproportionate focus on mistakes.

### Practical implications

The obtained findings have important practical applications for both researchers and practitioners. We believe that it is very important that researchers use adequate statistical procedures in addition to adequate instruments in order to obtain meaningful results. The statistical procedure (SAMM) used in the present study allows for testing complex models elaborating on empirically supported previous theories, such as those concerning the predictive relationships between irrational beliefs, perfectionism, and competitive anxiety. Practitioners may also benefit from the results of the present study. While REBT is a relatively new technique in sport (most research emerging post-2013; Jordana et al., 2020), the present study demonstrates its relevance to competitive anxiety. The results suggest that enabling athletes to gain insight into their irrational beliefs may help them better manage their perfectionism and competitive anxiety. A promising means to this end is the REBT procedure adapted for athletes (Turner and Bennett, 2018), which may be used in both individual and group settings. Practitioners should also be mindful that anxiety it is not always maladaptive (Ellis and DiGiuseppe, 1993) or related to poor performance (Craft et al., 2003), and thus, when working with athletes who report high anxiety, we need to understand the extent to which this anxiety is indeed maladaptive for the athlete. The distinction between adaptive and maladaptive emotion is a core consideration of REBT (e.g., Turner et al., 2018), which advocates the diminishment of maladaptive emotion and not adaptive emotion.

### Limitations and future directions

The current study has several limitations. It is a cross-sectional study exclusively involving Hungarian athletes, which does not allow for the exploration of true causality, nor does it allow for assessing the importance of cultural factors, while most related previous findings were obtained for North American and British samples. Turner et al. (2019a) found that elite athletes reported smaller depreciation beliefs, this result suggesting that the level of athlete may influence the emergence of irrational beliefs but not the relationship between irrational beliefs and the outcome variables. Based on the previous studies further research is needed to fully understand how athletes level influence irrational beliefs and it could be a further development of the current study to compare athletes at various levels in terms of irrational beliefs, perfectionism and competitive anxiety. Finally, in addition to perfectionism, other psychological constructs (e.g., negative automatic thoughts, fear of failure, early maladaptive schemas, self-determined motivation, stress mindset, and cognitive appraisal) may also influence the emergence of anxiety alongside irrational beliefs (e.g., Buschmann et al., 2018; Chadha et al., 2019; Turner et al., 2019b, 2022; Mansell, 2021), the exploration of which requires further study. Another important objective of future studies should be a longitudinal assessment of the effectiveness of psychological interventions (e.g., REBT) in regulating athletes’ competitive anxiety by restructuring irrational beliefs and perfectionism. The placement of perfectionism in REBT is yet to be fully conceptualized, and intervention research may highlight how practitioners can effectively challenge perfectionism within an REBT framework.

## Conclusion

The present study found that all types of irrational beliefs had an impact on competitive anxiety. Furthermore, the effects of several irrational beliefs on cognitive anxiety were partially mediated by adaptive and maladaptive perfectionism combined or by maladaptive perfectionism alone. These findings could suggest that an REBT intervention may help athletes effectively manage their maladaptive perfectionism and competitive anxiety.

## Data availability statement

The raw data supporting the conclusions of this article will be made available by the authors, without undue reservation.

## Ethics statement

The studies involving human participants were reviewed and approved by the Hungarian University of Sport Science Research Ethics Committee. Written informed consent to participate in this study was provided by the participants’ legal guardian/next of kin.

## Author contributions

RT: conceptualization, methodology, formal analysis, investigation, writing—original draft, and visualization. MT: conceptualization, resources, writing—review and editing, and supervision. TK: investigation and supervision. LT: conceptualization, investigation, and supervision. All authors contributed to the article and approved the submitted version.
